# Nudging Interventions on Alcohol and Tobacco Consumption in Adults: A Scoping Review of the Literature

**DOI:** 10.3390/ijerph20031675

**Published:** 2023-01-17

**Authors:** Mario Cesare Nurchis, Marcello Di Pumpo, Alessio Perilli, Giuseppe Greco, Gianfranco Damiani

**Affiliations:** 1 Department of Life Sciences and Public Health, Università Cattolica del Sacro Cuore, 00168 Rome, Italy; 2 Department of Woman and Child Health and Public Health, Fondazione Policlinico Universitario A. Gemelli IRCCS, 00168 Rome, Italy; 3 Azienda ULSS 6 Euganea, Regione Veneto, 35131 Padova, Italy

**Keywords:** alcohol, tobacco, nudge, prevention, chronic diseases, health policy

## Abstract

**Background:** The World Health Organization identified alcohol and tobacco consumption as the risk factors with a greater attributable burden and number of deaths related to non-communicable diseases. A promising technique aimed to modify behavioral risk factors by redesigning the elements influencing the choice of people is nudging. **Methodology**: A scoping review of the literature was performed to map the literature evidence investigating the use of nudging for tobacco and alcohol consumption prevention and/or control in adults. **Results:** A total of 20 studies were included. The identified nudging categories were increasing salience of information or incentives (IS), default choices (DF), and providing feedback (PF). Almost three-quarters of the studies implementing IS and half of those implementing PF reported a success. Three-quarters of the studies using IS in conjunction with other interventions reported a success whereas more than half of the those with IS alone reported a success. The PF strategy performed better in multi-component interventions targeting alcohol consumption. Only one DF mono-component study addressing alcohol consumption reported a success. **Conclusions:** To achieve a higher impact, nudging should be integrated into comprehensive prevention policy frameworks, with dedicated education sessions for health professionals. In conclusion, nudge strategies for tobacco and alcohol consumption prevention in adults show promising results. Further research is needed to investigate the use of nudge strategies in socio-economically diverse groups and in young populations.

## 1. Introduction

Tackling the burden of non-communicable diseases (NCDs) is a key public health goal [1]. In this regard, the World Health Organization identifies tobacco use, physical inactivity, alcohol consumption, and unhealthy diets as the principal risk factors for the development of NCDs [2]. Among these four, tobacco and alcohol consumption are responsible for the largest share of annual overall attributable deaths [2]. In particular, tobacco consumption was responsible for 8.71 million deaths in total in 2019 [3]. In 2000, an estimated 8.6 million people in the United States had an estimated 12.7 million smoking-attributable conditions [4]. The fraction of IHD cases attributable to smoking ranged from 13–33% in males and from 1–28% in females whereas the fraction of hemorrhagic strokes and ischemic strokes attributable to smoking ranged from 4–12% in males and from 1–9% in females, and from 11–27% in males and from 1–22% in females, respectively [5]. The burden of disease generated by tobacco consumption produced 229.77 million DALYs globally in 2019 [6]. Alcohol consumption is responsible for 3 million deaths in total each year, of which more than half are NCD-related [2]. In 2004, it was estimated that alcohol was responsible for 3.4% of the 35 million NCD-related deaths [7,8]. The burden of disease generated by alcohol consumption consisted of 207.31 (age-standardized) million disability-adjusted life years (DALYs) globally in 2019 [9].

In order to target these modifiable risk factors, many health promotion interventions are possible [10,11]. In the scientific literature, there are many examples of single- and multi-component as well as multi-level interventions available to professionals as effective public health tools [12,13,14].

Among these interventions, a promising and emerging technique in this regard consists in nudging. Thaler and Sunstein, in their groundbreaking work *Nudge: Improving Decisions about Health, Wealth, and Happiness* [15], define a nudge as “any aspect of the choice architecture that alters people’s behavior in a predictable way without forbidding any options or significantly changing their economic incentives”. The main aim is to modify behavior by redesigning the physical, social, and psychological environment that influences the decisions of people without restricting their freedom of choice [16,17,18,19].

Nudging theory, which emerged from the field of behavioral economics [20], was introduced as an innovative alternative strategy to traditional regulatory tools by adopting a new perspective—according to which, individuals do not always make the best decisions and choices about welfare and health [20]. Several studies include specific nudging techniques such as accessibility, presentation, using messages and pictures, technology-supported information, financial incentives, affecting the senses, and cognitive loading [21,22].

Inspired by behavioral economics, nudging techniques tap into cognitive biases and induce people to act and respect the interests and wellbeing of themselves and others [23]. Experts from various fields have analyzed the concept of nudging and investigated the implementation of different nudge policies [24]. Nudging interventions have been adopted in fields such as financial markets [25] and in education policies to produce effects on learning outcomes [26].

Nudging has been progressively applied to medicine [27] and public health [21,28]; in several countries, it has become increasingly innovative to help people to make propitious choices with a gentle approach to improve their health and wellbeing [29].

In the public health context, nudging has been used to address individual choices to produce desired behavioral changes, with the aim of enhancing population health outcomes. In particular, as it directly influences the behavior of individuals whilst preserving autonomy [20], nudging interventions can enable public health goals to be reached in a legitimate and democratic framework [20], whilst reducing the traditional use of regulation to control non-communicable diseases and their treatment [30].

With regard to the action upon the two aforementioned major chronic disease risk factors—alcohol and tobacco consumption—a recent systematic review by Blaga et al. [28] showed that offering incentives was one of the most common techniques used to change NCD health risk behaviors. There are already different analytic and synthetic pieces of evidence in the literature for each of these two designated risk factors [31,32,33,34]. In the current study, we propose a scoping review of the literature, addressing the use of nudging for the prevention and/or control of tobacco and alcohol consumption in adults in order to influence behaviors and clinical outcomes. The goal was to produce a useful literature synthesis for policy makers and public health and healthcare professionals for its implementation into practice.

## 2. Materials and Methods

### 2.1. Study Design and Search Strategy

Following the methodological framework proposed by Arksey and O’Malley [35] and refinements by the Joanna Briggs Institute [36,37], a scoping review of the literature was carried out to map the literature evidence investigating the use of nudging for the prevention and/or management of tobacco and alcohol consumption. The Preferred Reporting Items for Systematic Reviews and Meta-Analyses extension for Scoping Reviews (PRISMA-ScR) [38] was adopted in order to formulate the exhaustive search string needed to query the main scientific databases such as MEDLINE, EMBASE, Scopus, and Web of Science. The search was completed using the snowball search technique to retrieve additional studies or references from the included articles. The search string was built up by interpolating keywords such as “nudging”, “choice”, “behavior”, “smoking”, “alcohol”, and their synonyms through Boolean operators (i.e., AND/OR). The comprehensive search strategy is described in Table S2 in the Supplementary Materials. 

### 2.2. Study Selection

The eligibility criteria were defined according to the population, concept, and context (PCC) framework [36]. Particularly, the inclusion criteria were established as evidence involving a population of adults aged between 18 and 64 years old without any limitations in terms of gender or clinical conditions, describing the adoption of an intervention based on nudging techniques in community settings. In the definition of nudge-based interventions, the interpretation suggested by Thaler and Sunstein was applied [15]. In addition, the ladder of nudge interventions, provided by the Nuffield Council on Bioethics, was used as a guideline for identifying the main categories of nudge-based interventions in healthcare [39,40].

Given the nature of the intervention, the considered community settings were primary care clinics, community centers, and online platforms. 

The inclusion of studies was also restricted to the article type (i.e., primary research articles), language (i.e., English), and availability of full texts. Table 1 summarizes the inclusion and exclusion criteria.

Both the first round of screening by titles and abstracts and the second one by full texts were conducted by four independent authors. Any potential disagreements were resolved by a fifth researcher.

### 2.3. Data Extraction

An electronic data extraction form was set up to collect, for each included paper, information about the general characteristics of the study (i.e., name of the author, country, year of publication, and title) as well as the population, setting, type of nudge-based intervention, and outcomes achieved. The data extraction process was carried out by four independent authors. 

### 2.4. Nudge-Based Intervention Classification

The nudge-based interventions described by each included study were classified according to the categorization available in the seminal work by Thaler and Sunstein and already reported in the scientific literature [41]. The reference categories were based on the implementation techniques available to the choice architect and included the following: increasing salience of information or incentives (IS); understanding mapping (UM); default choices (DF); providing feedback (PF); error reduction (ER); and structuring complex choices (SC) [15]. The IS strategy entails the provision of information to expand the prominence, noticeability, or conspicuousness of information related to the choice alternatives (e.g., text, color, and alerts to increase decision option salience). The UM choice architecture eases the relationship between the choice alternatives available to a decision-maker and the related outcomes (e.g., flowcharts or decision trees summarizing the main points). The DF strategy implies that, for a given choice, there is a default option obtainable if the chooser does nothing. This option requires the path of least resistance (e.g., opt-out approach of a default order set). The PF strategy allows decision-makers to be informed about the outcomes of their behaviors, thus steering future behavior (e.g., feedback/warning systems to improve performance). The ER strategy involves the adoption of prompts to minimize common mistakes (e.g., prompts or forcing functions). The SC strategy entails the splitting-up of choice alternatives and attributes to simplify choice strategies (e.g., the use of categories to structure choices). Structuring a choice may help individuals to learn so that they can make better choices [42].

### 2.5. Data Synthesis

A preliminary descriptive synthesis was conducted tabulating a summary of the general characteristics of the included studies. Moreover, a narrative synthesis was performed considering several types of nudge-based interventions in healthcare for the prevention and/or management of tobacco and alcohol consumption and the relative observed success rate. The success rate was intended to describe the final outcome of the investigated intervention by analyzing either the reported *p*-values and confidence intervals for the quantitative studies or the adopted conceptual frameworks for the qualitative studies.

## 3. Results

### 3.1. Study Selection and Characteristics 

**Figure 1:**
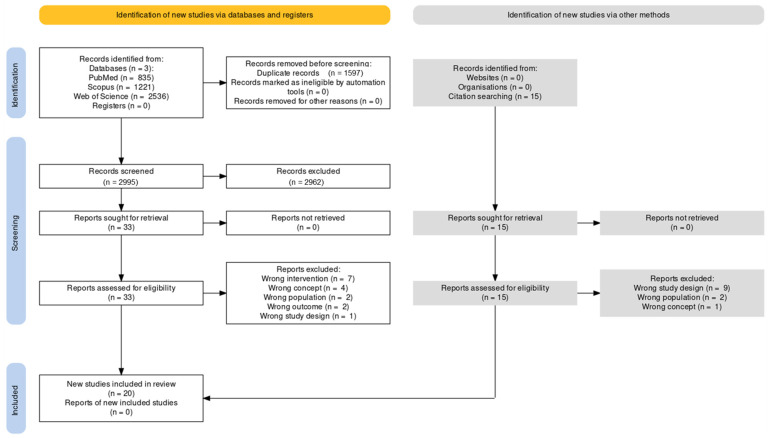
PRISMA-ScR flow diagram of the selection process.

Overall, after data de-duplication, the search strategy resulted in 3051 records from all the scientific databases. Of the total number, after the two rounds of screening (i.e., titles, abstracts, and full texts), 3031 studies were screened out due to an incompatibility with the inclusion criteria (e.g., wrong intervention, no outcome measurement, wrong study design, or publication type). As a result, 20 studies [43,44,45,46,47,48,49,50,51,52,53,54,55,56,57,58,59,60,61,62] were included in the review. The full selection process is shown in Figure 1.

Of the 20 studies in the final review, 10 (50%) [49,50,53,54,56,57,58,60,61,62] were conducted in the USA, 6 (30%) in the UK [45,46,48,52,55,59], 2 (10%) in South Africa [44,51], and 2 in Bangladesh (10%) [43,47]. 55% of the included studies focused their interventions on smoking cessation [43,45,47,50,54,56,57,58,59,61,62], 35% on alcohol drinking cessation [46,48,49,52,53,55,60], and 10% targeted both risk factors [44,51]. Table S1 in the Supplementary Materials provides further details on the characteristics of the studies. 

Furthermore, seven (35%) studies [44,45,51,53,54,57,61] involved the use of a nudge strategy as part of a multi-component intervention whereas the remainder (65%) adopted nudge strategies as single-component interventions [43,46,47,48,49,50,52,55,56,58,59,60,62]. 

### 3.2. Classification of Nudge Interventions for Smoking and Alcohol Cessation

The use of several nudge types that were adopted in the included studies as interventions was assessed and classified according to the classification by Thaler and Sunstein as well as evidence available in the scientific literature.

The present review showed that the retrieved articles on the prevention of alcohol and tobacco consumption examined the use of three out of six nudge strategies (i.e., IS, PF, and DF).

*Increasing salience of information or incentives* (*IS*). The most popular nudge strategy adopted was IS, which aims to increase the salience of information or incentives to steer the attention of individuals to the desired choice. Fourteen of the included studies implemented the IS strategy. Of the fourteen studies, ten used IS alone (i.e., a single-component intervention) whereas the remaining four articles adopted the hitherto-cited choice architecture in combination with other interventions (i.e., a multi-component intervention). 

*Providing feedback* (*PF*). According to the included evidence, the second nudge strategy adopted was PF, which implies the provision of information to individuals on the consequences of their behavior. Four of the included articles adopted the PF strategy. Out of the four studies, one-half used PF alone and the other half used a combination with other interventions. 

*Default choices* (*DF*). Default choices, the nudge strategy that puts the choice setting along the path of least resistance, was another strategy examined. Its adoption was investigated in two of the studies. Of the two studies, one used DF alone whilst the other used DF in conjunction with other interventions. 

### 3.3. Nudge Intervention Success Rate

Table 2 shows a breakdown of the success rates of the study interventions according to the topic investigated (i.e., tobacco and/or alcohol) and the setting chosen (i.e., community, clinical, or web-based).

Overall, of the fourteen studies using IS as choice architecture strategy either alone or in combination with other interventions, most reported a success; however, four out of fourteen reported a lack of success. When used in conjunction with other interventions (i.e., four articles), the IS strategy led to successful results in three studies, whereas, when taken alone (i.e., ten articles), successful findings were reported in six studies.

Of the four studies implementing PF as a nudge strategy, half described successful findings and the other half reported a lack of success. Particularly, when used in combination with other interventions, it seemed that the PF strategy performed better in preventing the risk factors associated with alcohol consumption with respect to using it as a stand-alone strategy, which, on the contrary, failed to achieve successful results for preventing the risk factors related to tobacco consumption.

Only one study reported successful results addressing alcohol consumption adopting the DF nudge strategy alone. The other study, focusing on tobacco consumption and implementing DF jointly with additional interventions, failed to achieve significant findings.

## 4. Discussion

The present review found that the IS nudge strategy was the most adopted among the included articles. Furthermore, the review showed a higher overall success rate for the studies adopting the IS nudge strategy both for alcohol and tobacco consumption prevention.

Considering the nature of the IS choice architecture, the review results were endorsed by evidence in the scientific literature highlighting the larger effectiveness of image-based warning signs at communicating risk and discouraging dangerous activities [63]. Particularly, the higher effectiveness associated with this type of choice architecture for tobacco control may derive from the increased awareness of the health risks related to tobacco consumption [64,65]. In line with this, the WHO issued Article 11 of their *Framework Convention on Tobacco Control* (WHO FCTC) for regulating the use of health-warning graphic labels depicting the health risks and potential health effects caused by smoking [66]. For alcohol consumption, scientific evidence also confirmed the review findings, highlighting that image and text warning labels discourage the consumption of alcoholic beverages [67,68]. As with tobacco consumption, in its last snapshot series on alcohol control policies and practice, the WHO recommended labeling alcoholic beverages to increase awareness and guarantee that individuals make informed decisions [69]. This category of nudge also fits within the scope of the EAST Framework on Behavior Change [70].

The underlying mechanism lies in negative emotional stimuli such as fear, disgust, discomfort, and worry, which are triggered by warnings [67]. Other aspects accounting for its relevance include the wide public support that it seems to enjoy as well as costs being borne by companies, not governments, its ability to affect a very large population, and its proneness to modifications and improvements [71]. 

Staying in the area of prevention, in addition to tobacco and alcohol, this type of intervention also appears to be effective in reducing sugary drink purchases, thus tackling the obesity burden [72]. Furthermore, scientific evidence also described the application of behavioral nudges displayed as posters as an inexpensive, easy, and effective measure in the promotion of hand hygiene for reducing infections in healthcare settings [73]. In light of the above considerations, salience nudges may potentially acquire a variety of public health applications. 

According to our findings, another frequent nudge was the PF type. We found that two out of the four studies deploying this type of nudge intervention achieved positive outcomes. The literature suggests that nudges on information and feedback might actually lead to weaker impacts, supposedly caused by distractions or information overload compared with more powerful tools [74]. Attention should also be paid to the intervention timing.

The joint reading of the study findings led to a first main implication, which was the support in informing programs and policies for health prevention. Indeed, evidence-based nudges can be viewed as useful public health policy tools, despite the complex environment surrounding and affecting people’s health decisions [75]. To ensure a greater impact, it may be appropriate to integrate nudges into a comprehensive prevention policy framework [1,70]. Interestingly, previous research also showed an acceptable level of citizen approval [76]. Furthermore, it is of paramount importance to focus on the educational lever in developing nudge-based interventions. Health professionals need to be educated to set up targeted as well as more effective and efficient nudge-based interventions for health prevention.

The present study must be assessed considering its main limitations and strengths. First, a limited number of articles was included because most of the retrieved occurrences did not adopt nudge-based interventions. However, a robust process comprising the search strategy, study selection with strict inclusion criteria, and data synthesis was conducted to improve the study relevance. Moreover, there was heterogeneity among the nudge strategy categories identified based on several interventions. Nevertheless, the heterogeneity could be explained by the structural and methodological diversity among the included manuscripts. In addition, a quality assessment of each analyzed paper was not conducted; thus, a potential risk of bias was not considered. However, no formal quality assessment is required for scoping reviews [77]. A noteworthy structural limit refers to the criticism towards the nudge approach on which the primary studies included in the present review were based. Indeed, according to Maier et al. after controlling for a publication bias, the nudge approach may be not an effective tool for behavior changes [78].

Further studies are required to evaluate nudge-based interventions for alcohol and tobacco consumption prevention by conducting, from a multi-dimensional and multi-professional perspective, tailored assessments for each domain (e.g., cost and economic, organizational, ethical, legal, and societal issues) characterized by the Health Technology Assessment process [79]. The durability of results ought to be assessed thoroughly in future studies, as well [80]. As shown in our study, a few nudge strategies also remain under-researched; most of the studies included fitted into the IS or PF category. Future research should take advantage of this literature gap and fill it.

Particularly, investigating the equity dimension in applying nudge strategies to socio-economically diverse groups is of paramount importance for the development of tailored prevention campaigns. Moreover, additional research is required to assess nudge-based interventions for health prevention in young populations. 

## 5. Conclusions

Based on the available information, the present review documented a large dominance of IS and PF nudges applied to tobacco and alcohol consumption prevention in adults, with mixed results on their effectiveness.

It is important to identify the specific outcome measures in a comprehensive prevention framework [1,70] to perform progress monitoring to evaluate and implement each strategy and steer new decisions and programs. Further studies should be conducted over longer periods of time and include established prevention interventions as comparators.

Given the importance of the burden of disease generated by tobacco and alcohol, researchers, professionals, and stakeholders should consider and evaluate combined strategies and their benefits, and assess their impacts in the healthcare context.

## Figures and Tables

**Table 1:** Inclusion and exclusion criteria.

PCC	Inclusion Criteria	Exclusion Criteria
Population	Population of adults aged between 18 and 64 years old without any limitations in terms of gender or clinical conditions	Pediatric population
Concept	Interventions based on nudge strategies	–
Context	Community settings	–

PCC: population, concept, and context.

**Table 2:** Nudge intervention success rate based on the topic and setting.

		Total	Successful		Unsuccessful	
Risk Factor Targeted	Setting	Number	Number	%	Number	%
Tobacco	Community	8	5	62.5	3	37.5
	Online	3	1	33	2	67
Alcohol	Community	2	1	50	1	50
	Online	1	1	100	–	–
	Primary care clinics	2	1	50	1	50
	Mixed	1	1	100	–	–
	Not reported	1	1	100	–	–
Tobacco andalcohol	Primary care clinics	2	1	50	1	50

## Data Availability

Data is available upon request to the corresponding author.

## Supplementary Materials

The following supporting information can be downloaded at: https://www.mdpi.com/article/10.3390/ijerph20031675/s1, Table S1: Summary general characteristics of the included studies. Table S2: Search Strategy.
