# Supplementary material for: Nudging Interventions on Alcohol and Tobacco Consumption in Adults: A Scoping Review of the Literature
Source: Int J Environ Res Public Health. 2023 Jan 17;20(3):1675. doi: 10.3390/ijerph20031675 (PMC9913966; doi:10.3390/ijerph20031675)
Supplement: Supplementary file 1 [file ijerph-20-01675-s001.zip › ijerph-2118793-supplementary.pdf]

Table S1. Summary general characteristics of the included studies

Author, Year, Country	Nudge category	Risk factor targeted	Study design	Complexity of intervention	Mean age	Sample size	Intervention	Comparator
Fakir MSA, 2022, Bangladesh [43]	Increasing salience of information or incentives (IS)	Tobacco	Randomized field experiment	Single component	39	1215	LT: recording the total smoking and SLT consumption, and its estimated monetary cost, in a pictorial logbook daily.<br>PT: placement of two graphical posters in the sleeping quarters of the participating household head and the spouse. These posters were visual reminders of the health costs of smoking.	No comparator
Louwagie G, 2022, South Africa [44]	Increasing salience of information or incentives (IS)	Tobacco and alcohol	RCT	Multi component	39	574	Three brief motivational interviewing (MI) sessions, augmented with short message service (SMS) messages, targeting medication adherence, alcohol use and tobacco smoking delivered by lay health workers (LHWs).	Usual care
Hasin DS, 2022, USA [53]	Providing feedback (PF)	Alcohol	RCT	Multi component	48	114	MI, HealthCall (an application that uses smartphone technology to provide brief, continued between session) patient engagement), health education	MI-only vs MI plus HealthCall vs health education-only
Clarke N, 2021, UK [52]	Increasing salience of information or incentives (IS)	Alcohol	Randomized field experiment	Single component	40	399	Health warning labels (HWLs) on alcohol drinks to reduce alcohol drinking	No HWL intervention
Tripp HL, 2021 USA [57]	Increasing salience of information or incentives (IS)	Tobacco	Cross-sectional study	Multi component	41	512	In each experimental message group, participants were exposed to three messages (health, cost, and aesthetics, respectively)	No intervention
Naudè GP, 2021 USA [56]	Increasing salience of information or incentives (IS)	Tobacco	Randomized field experiment	Single component	36	622	Experiment 1: effects of episodic future thinking (EFT) and graphic warning labels (GWL) on delay discounting.	No intervention

							Experiment 2: solely the effects of episodic future thinking on delay discounting and operant demand	
McRobbie HJ, 2020, UK [45]	Default choices (DF)	Tobacco	RCT	Multi component	44	235	The Structured Planning and Prompting Protocol and interactive text messages	Usual care plus static text messages
Langfield T, 2020, UK [46]	Increasing salience of information or incentives (IS)	Alcohol	Case report	Single component	22	312	Study 1: Use of straight-sided and outward-sloped tumblers<br>Study 2 & 3: Use of outward-sloped martini coupes	Study 1: Use of straight-sided glasses<br>Study 2 & 3: Use of straight-sided wine flutes
Fakir MSA, 2020, Bangladesh [47]	Providing feedback (PF)	Tobacco	RCT	Single component	44	461	Two visual warning posters, detailing the health effects of tobacco on oneself and external actors, to be hung inside the household	No comparator
A González-Roz, 2020, USA [54]	Increasing salience of information or incentives (IS)	Tobacco	Retrospective cohort study	Multi component	49	305	Cognitive–behavioral treatment (CBT) plus CM (Contingency management)	CBT
Clarke N, 2021, UK [55]	Increasing salience of information or incentives (IS)	Alcohol	Randomized field experiment	Single component	50	6024	Image-and-text, text-only and image-only HWL (Health warning labels)	Text-only
DeHart WB, 2019, USA [50]	Providing feedback (PF)	Tobacco	Randomized field experiment	Single component	37	157	Use of cognitive biases in public health narratives	Usual care
Louwagie GM, 2020, South Africa [51]	Providing feedback (PF)	Tobacco and alcohol	Retrospective observational study	Multi component	40	45	Motivational Interviewing (MI), a patient-centered counseling approach to address multiple behavioral problems, augmented with a Short Messaging Service (SMS) Program	No comparator
Martinetti MP, 2019, US [60]	Default choices (DF)	Alcohol	Cross-sectional study	Single component	20	268	Additional choice	No comparator
Kersbergen I, 2018, UK [48]	Increasing salience of	Alcohol	Randomized field experiment	Single component	30	278	Use of reduced (by 25%) glasses for alcohol consumption	Use of standard glasses for alcohol consumption

	information or incentives (IS)							
Lewis MA, 2018, USA [49]	Increasing salience of information or incentives (IS)	Alcohol	Randomized field experiment	Single component	23	48	12 TMs over the next 2 weeks (2 messages a day, 3 days a week) on the days they indicated in the baseline survey as their typical drinking days. Messages included PBS presented in various ways including testimonials, tips, tailored or personalized messages, and “mocktail” recipes	No comparator
Hall MG, 2018, US [58]	Increasing salience of information or incentives (IS)	Tobacco	RCT	Single component	40	2149	Pictorial warning	Text-only warning
Rash CJ, 2018, USA [61]	Increasing salience of information or incentives (IS)	Tobacco	RCT	Multi component	45	70	Standard care plus contingency management	Standard care
Stothart G, 2016, UK [59]	Increasing salience of information or incentives (IS)	Tobacco	Cross-sectional study	Single component	23	40	Pictorial warning	Text-only warning
Brewer NT, 2016, USA [62]	Increasing salience of information or incentives (IS)	Tobacco	RCT	Single component	39	2149	Pictorial warning	Text-only warning

Abbreviations: RCT, Randomized Controlled Trial; MI, Motivational Interviewing; CBT, Cognitive–behavioral treatment; CM Contingency Management; HWLs, Health warning labels; EFT, Episodic Future Thinking; GWL, Graphic Warning Labels.

Table S2. Search Strategy

Database	Search	Results
PubMed	(nudge* OR nudg* OR behav* econom* OR bias, cognition (MeSH) OR "behavioral intervention" OR "behavioural intervention" OR "behavioral strategy" OR "behavioural strategy") AND (alcohol* OR ethanol OR drink* OR "binge drinking" OR "Alcoholic Intoxicat*" OR Alcoholism OR addiction OR liquor* OR beer* OR wine* OR spirit* OR drunk* OR smok* OR nicotine OR anti-smok*)	835

Database	Search	Results
Scopus	TITLE-ABS-KEY (nudge* OR nudg* OR {behav* econom*} OR {bias, cognition} OR {behavioral intervention} OR {behavioural intervention} OR {behavioral strategy} OR {behavioural strategy}) AND TITLE-ABS-KEY(alcohol* OR ethanol OR drink* OR {binge drinking} OR {Alcoholic Intoxicat*} OR Alcoholism OR addiction OR liquor* OR beer* OR wine* OR spirit* OR drunk* OR smok* OR nicotine OR anti-smok*)	1221

Database	Search	Results
WoS	TS=(nudge* OR nudg* OR "behav* econom*" OR "cognitive bias" OR "behavioral intervention" OR "behavioural intervention" OR "behavioral strategy" OR "behavioural strategy") AND TS=(alcohol* OR ethanol OR drink* OR "binge drinking" OR "Alcoholic Intoxicat*" OR Alcoholism OR addiction OR liquor* OR beer* OR wine* OR spirit* OR drunk* OR smok* OR nicotine OR anti-smok*)	2536
